# Interferon-Based Therapeutics in Cancer Therapy: Past, Present, and Future

**DOI:** 10.3390/ijms262311679

**Published:** 2025-12-02

**Authors:** Kristina Vorona, Anastasia Ryapolova, Olesya Sokolova, Alexander Karabelsky, Roman Ivanov, Vasiliy Reshetnikov, Ekaterina Minskaia

**Affiliations:** Translational Medicine Research Center, Sirius University of Science and Technology, 1 Olympic Avenue, 354340 Sochi, Russia

**Keywords:** interferon, IFN, interferon type, anti-cancer therapy, antitumor therapy

## Abstract

Interferons (IFNs) are well-known immunostimulants involved in both innate and adaptive immune responses. These multifunctional proteins mediate an early antiviral response and have pronounced immunomodulatory and antiproliferative properties. Due to their potency, IFNs have been used not only in the treatment of viral infections but also various other diseases. However, the use of IFNs in antitumor therapy has been limited by the frequent severe side effects, which reduced their appeal for the treatment of cancer. In this review, we focused on current data on recombinant IFNs used for anticancer therapy, as well as the development of promising IFN-based gene therapy approaches, with a focus on their safety and therapeutic efficacy. We also highlighted various types of IFNs and their application niches in the treatment of not only cancers in combination therapy but also of certain rare diseases. Taken together, this review improves our understanding of the existing IFN applications in cancer therapy, the disadvantages of using IFNs, and possible approaches for their improvement.

## 1. Introduction

Cancer is a disease caused by mutations or epigenetic changes in the body’s cells, which leads to their uncontrolled growth and division, followed by the formation of a tumor and possible metastasis [1]. Despite the intensive development of new approaches in cancer therapy, including immune checkpoint inhibitors, bispecific T-cell engagers (BiTE), oncovirotherapy, and chimeric antigen receptor T-cell therapy (CAR-T), the number of cancer patients is steadily increasing [2]. According to global cancer statistics, in 2022, almost 20 million people had cancer, and about 10 million died from it [3]. The forecasts predicting the number of new cases are concerning: 35 million people with cancer are expected to be diagnosed by 2050 [3].

Interferons (IFNs) occupy one of the central places among well-known anticancer agents. After their discovery more than 50 years ago, the FDA approved the first recombinant IFNa2b (Intron A^®^)-based drug in 1986 (almost 30 years ago), which was used for the treatment of melanoma, kidney cancer, follicular lymphoma, and various types of leukemia [4]. IFNs are involved in antiviral immunity and the fight against cancer cells [5,6]. Type I interferons (IFN-I) include 13 subtypes of IFN-α, as well as IFN-β, IFN-ω, IFN-ε, and IFN-κ, while IFN-γ is a type II interferon. Therapies based on IFN-α and IFN-γ have a pronounced antitumor effect on different types of cancer [5,6]. The golden era of recombinant IFN-based drugs occurred in the 1980s and 1990s [5]. Nevertheless, a wide range of possible side effects, demonstrated in recent years, has limited the application of therapies based on recombinant IFNs.

Specifically, many studies analyzing the potency of IFN-α as a therapeutic agent for cancer treatment discovered problems with its short half-life, low efficacy, and numerous, often intolerable, side effects [7]. In addition to methods for modifying the molecule itself, high doses of IFN-α are also used to achieve better efficacy; however, this too may lead to numerous side effects [8]. Due to the presence of IFN-α receptors on the surface of almost every cell in the body, off-target effects occur, causing many adverse reactions [9,10]. Furthermore, progress in immune- and targeted therapies for malignant neoplasms (especially the development of effective drugs based on immune checkpoint inhibitors (ICIs) and BiTE has dampened the widespread use of recombinant IFNs for the treatment of oncological diseases.

Despite the development of new innovative drugs and approaches, key treatment strategies for many cancer types are still radiation therapy, chemotherapy, hematopoietic stem cell transplantation, hormone therapy, antibody-drug conjugates, and photodynamic therapy [2]. In this context, the fundamental mechanisms of IFN action also have the potential to find their place in oncology, both in combination therapies and for the treatment of certain rare cancers. In this review, we analyze the advantages and disadvantages of approved IFN-based drugs and discuss the promising results of certain IFN-based clinical trials.

## 2. The Innate Antiviral Response. Signaling Pathways for Type I IFN Production

**Figure 1:**
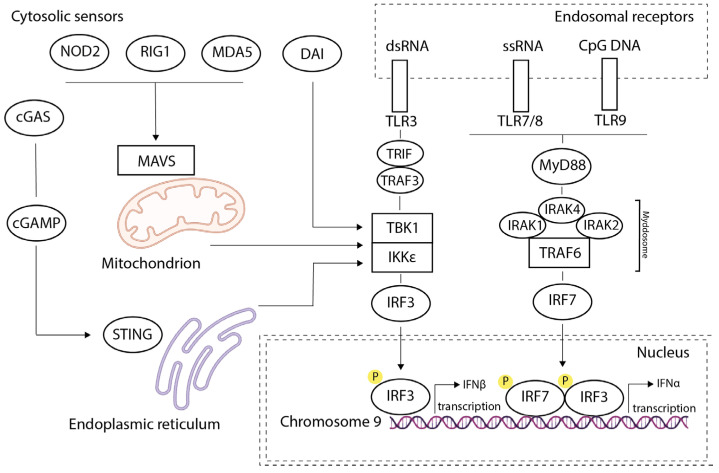
PAMPs and DAMPs are recognized by both endosomal receptors (TLR3, TLR7/8, TLR9) and cytosolic sensors (NOD2, RIG-1, MDA5, DAI, and cGAS). Their activation triggers signaling cascades leading to phosphorylation of the transcription factors IRF3 and IRF7. Phosphorylated IRF3/IRF7 are transported to the nucleus, where they bind to the promoters of type I interferon genes, stimulating their expression.

The innate immune system detects foreign microorganisms within the body and activates mechanisms to eliminate potentially dangerous infectious agents [11]. When foreign microorganisms enter cells, complex interactions between the pathogen and the host occur; microorganisms express several low-molecular-weight motifs known as Pathogen-Associated Molecular Patterns (PAMPs) or cause damage that leads to the release of Damage-Associated Molecular Patterns (DAMPs) in the host cell’s cytoplasm [12,13]. Mannose-rich oligosaccharides, peptidoglycans, and lipopolysaccharides of the bacterial cell wall, as well as foreign DNA and RNA, can act as PAMPs. These motifs are evolutionarily conserved, which makes them excellent targets for the host’s innate immune system [14]. DAMPs are released following injury or stress and include extracellular adenosine triphosphate (ATP), cholesterol crystals, sodium urate/calcium pyrophosphate dihydrate, glucose, amyloid β, and hyaluronan [15]. PAMPs and DAMPs are recognized by evolutionarily conserved host receptors known as pattern recognition receptors (PRRs), which are encoded by the germline and are important elements of the innate immune system [16]. Following the recognition of PAMPs and DAMPs, multiple immune reactions are triggered, leading to the synthesis of inflammatory cytokines, chemokines, and IFN-I [13,17,18] (Figure 1).

PRRs include Toll-like receptors (TLRs), C-type lectin receptors (CLRs), RIG-I-like receptors (RLRs), NOD-like receptors (NLRs), AIM2-like receptors (ALRs) [12,19,20], and other cytosolic sensors. TLRs and CLRs are membrane-bound receptors that recognize PAMPs and DAMPs on the cell surface and inside endosomes. ALRs, NLRs, and RLRs, as well as cytosolic sensors, recognize PAMPs and DAMPs that have entered the cytoplasm due to infection by pathogens or damage to host cell organelles [12].

TLRs are located in the endosomal membranes of many immune cells, such as macrophages, dendritic cells (DCs), and B cells, where they monitor the presence of bacterial and viral nucleic acids in the lumen of lysosomes and endosomes [21]. Ten members of the TLR family have been identified in humans, five of which—TLR3, TLR7, TLR8, TLR9 are involved in the recognition of pathogenic nucleic acids. TLR3 recognizes dsRNA, TLR7 and TLR8—ssRNA, while TLR9 recognizes DNA [22]. The receptors in this family function via two main signaling pathways: TLR7, TLR8, and TLR9 mediate the activation of MyD88, whereas TLR3 activates TRIF [21,23,24,25]. MyD88 and TRIF transmit signals that activate the transcription of genes encoding IFN-I (Figure 1).

The promoter region of IFN-I genes, located before the transcription initiation site and the TATA box, contains several promoter regulatory domains that are activated by two interferon regulatory factors (IRFs): IRF-3 and IRF-7. Upon activation, IRF-3 and IRF-7 translocate to the nucleus, bind to the promoter, and initiate IFN-I transcription. Furthermore, small differences in the VRE sequences of different IFN-I genes affect the binding affinity of IRF-3 and IRF-7, which may explain some temporal and quantitative differences in gene expression [26].

In mammalian cells, there are two main recognition pathways for cytosolic nucleic acids (Figure 1): the cGAS–STING pathway (cyclic GMP–AMP synthase–stimulator of interferon genes) and the RLR–MAVS pathway (RIG-I–like receptors–mitochondrial antiviral-signaling protein), which recognize cytosolic DNA and RNA, respectively [21,27].

RLRs (RIG-I, MDA5, and LGP2) recognize viral double-stranded RNA. Upon binding to viral RNA, RIG-I and MDA5 activate a common downstream adapter molecule, MAVS. Activated MAVS then recruits a variety of signaling molecules, including TRAFs, TBK1, and IRF3/7, leading to the enhanced transcription of type I interferons and other pro-inflammatory cytokines [28,29,30,31,32,33].

Cyclic GMP–AMP synthase (cGAS) recognizes double-stranded DNA. When cGAS binds to DNA, it synthesizes cyclic GMP–AMP (cGAMP), which in turn binds to and activates STING. The activation of STING triggers a downstream transcriptional program via IRF3 and nuclear factor κB (NF-κB), resulting in the increased production of IFN-I, proinflammatory cytokines, and chemokines [21,34].

In addition, other sensors such as IFI16, DDX41, DNA-PK, MRE11, and DAI can act as cytosolic sensors of foreign nucleic acids [21]. Their mechanisms of action are also associated with the activation of STING and the triggering of downstream signaling pathways, leading to the phosphorylation of IRF3 and IRF7. Following phosphorylation, IRF3 and IRF7 translocate to the nucleus, bind to the promoters of IFN-I genes, and activate their transcription.

## 3. The IFN Family: Canonical and Non-Canonical Signaling

**Figure 2:**
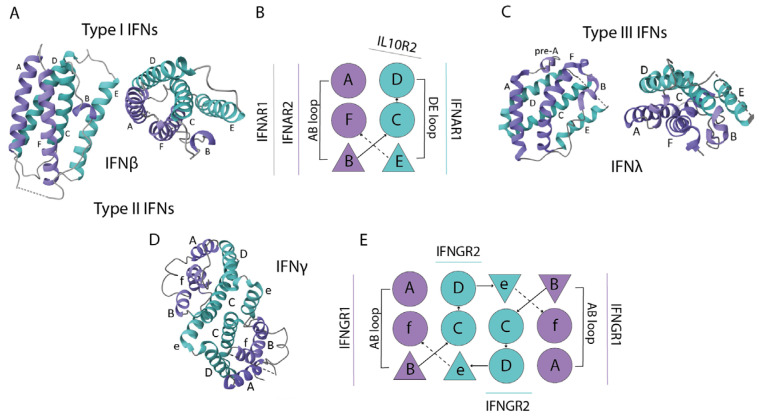
Structures of type I, type II, and type III IFNs: (**A**) Structure of type I interferons, illustrated by the crystal structure of human IFN-β at 2.2-Å resolution, PDB ID—1AU1; (**B**) Diagram of type I and type III IFNs secondary structural components: α-helix (A–F); (**C**) Structure of type III interferons, illustrated by the crystal structure of human IFN-λ3 at 2.8-Å resolution, PDB ID—3HHC; (**D**) Structure of type II interferons, illustrated by the crystal structure of human IFN-γ at 2.9-Å resolution, PDB ID—1EKU; (**E**) Diagram of type II IFN secondary structural components: α-helix (A–F).

Interferons are central to the innate immune system. They are cytokines that perform immunoregulatory functions, mainly related to antiviral reactions, adaptive immunity, and antiproliferative effects on immune and somatic cells [35]. In humans, IFNs are classified into three types (I–III) depending on the type of receptor through which the signal is transmitted (Figure 2).

Type I IFNs interact with the IFNAR receptor and comprise up to 17 subtypes that share 20–60% sequence homology [36,37]. These include IFN-β, IFN-ω, IFN-ε, IFN-κ, and 13 subtypes of IFN-α. IFN-I plays a key role in cellular antiviral and antiproliferative responses and is also crucial for regulating both innate and adaptive immunity [38,39,40].

In contrast to the multiple type I IFNs, type II IFN (IFN-II) is represented by a single cytokine, IFN-γ. It is essential for cell-mediated immunity, activating macrophages and promoting the development of CD4^+^ Th1 cells and cytotoxic CD8^+^ T cells [41,42,43,44,45,46,47,48,49,50]. IFN-γ binds to a specific heterodimeric receptor composed of IFNGR1 and IFNGR2 subunits [51]. Its production is limited to specific cell types, including CD4^+^ Th1 cells, cytotoxic CD8^+^ T cells, and natural killer (NK) cells. The interaction of IFN-γ with its receptor complex initiates a signaling cascade via JAK kinases and STAT transcription factors. Activation of the JAK-STAT pathway coordinates key cellular functions, such as immune activation, cell proliferation, and apoptosis, and exerts multifaceted effects on tumor cell growth, exhibiting both anti-tumor and pro-tumor activities [52].

Type III IFNs (IFN-III) include IFN-λ1, IFN-λ2, IFN-λ3 (also known as IL-29, IL-28A, and IL-28B, respectively), and IFN-λ4 [53,54,55]. They signal through the IFNLR receptor, a heterodimer consisting of the IFNLR1 (or IL-28Rα) subunit and the IL-10Rβ subunit, the latter of which is shared with other cytokine receptors (e.g., for IL-10, IL-22, IL-24, and IL-26) [56]. Although the intracellular signaling pathways of IFN-I and IFN-III are similar, the expression of the IFNLR receptor is significantly more restricted than the ubiquitous IFNAR receptor. The IFNLR receptor is expressed primarily on epithelial cells and certain immune cells (e.g., neutrophils, DCs) [40,41,57]. This specific expression profile defines IFN-λ’s role as a first-line defense cytokine at mucosal surfaces against viruses, such as in the intestinal [58,59,60] and pulmonary epithelia [61] (Figure 2).

The IFN-I receptor, IFNAR, consists of IFNAR1 and IFNAR2 subunits. The IFNAR2 subunit plays the primary role in ligand binding, exhibiting high affinity for IFNs in the nanomolar range even in the absence of IFNAR1. In contrast, IFNAR1 has a much lower affinity, with a dissociation constant in the micromolar range [62,63].

The canonical IFN-I signaling pathway is a series of biochemical events involving JAK/STAT proteins that control transcriptional activation of IFN-stimulated genes (ISGs) involving the Jak1 and Tyk2 kinases. These kinases, in turn, phosphorylate STAT1 and STAT2, which form heterodimers, and STAT1 and STAT3, which form homodimers. The phosphorylated STAT1-STAT2 heterodimer then associates with IRF9 to form the ISGF3 complex, which translocates to the nucleus to initiate the transcription of IFN-stimulated genes (ISGs). These ISGs are responsible for the antiviral and anti-proliferative states. Meanwhile, the STAT1 homodimer is associated with a pro-inflammatory response, mediated by binding to gamma-activated sequences (GAS), and the STAT3 homodimer indirectly inhibits inflammatory gene expression, restraining pro-inflammatory responses. Notably, the activation of different ISGs requires different concentrations of IFNs, reflecting their varying affinities for the IFNAR receptor [63,64,65].

**Figure 3:**
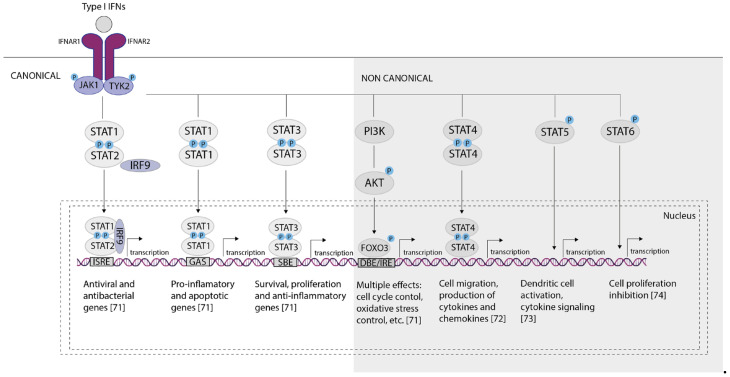
Canonical and non-canonical IFN signaling. Type I IFNs initiate signaling by binding to the common IFNAR receptor, forming a ternary complex with its IFNAR1 and IFNAR2 subunits. This triggers the sequential activation (phosphorylation) of the receptor-associated Jak1 and Tyk2 kinases. These kinases then phosphorylate downstream targets, including STAT proteins, as well as components of the MAPK and PI3K pathways. The canonical signaling pathway involves the formation of the pSTAT1, pSTAT2, and IRF9 (ISGF3) trimer, which moves to the nucleus, binds to ISRE, and triggers transcription of ISGS responsible for the antiviral and antibacterial response. There are many non-canonical signaling pathways, one of which is the formation of pSTAT1 homodimers and binding to GAS, followed by transcription of pro-inflammatory and pro-apoptotic genes. Non-canonical signaling also includes phosphorylation and formation of homo- and heterodimers of STAT3, STAT4, STAT5, and STAT6. Interactions with MAPK (mitogen-activated protein kinase) and PI3K (phosphoinositide 3-kinase) kinases can also occur, leading to various effects in the cell.

Non-canonical IFN-I signaling involves JAK/STAT-independent signaling pathways. They are similarly activated by IFNs binding to the extracellular regions of the dimeric IFNAR1 and IFNAR2 complexes, leading to JAK1/TYK2 activation, but diverge from that point, specifically not involving STAT activation by the JAKs, such as interactions with other STAT family members, and engagement of the MAPK or PI3K pathways [66,67,68,69,70] (Figure 3). At the same time, depending on which signaling pathway is activated, they are capable of activating the transcription of various ISG [71,72,73,74].

Type I IFNs can activate signaling molecules such as STAT1, STAT2, STAT3, STAT5, STAT4, and STAT6 in lymphocytes [71,75,76]. Non-canonical IFN-I signaling pathways primarily activate ISGs that exhibit graded, rather than switch-like, dose responses and require higher IFN concentrations to achieve maximum expression [66,77]. These non-canonical pathways promote the expression of chemokines and cytokines that regulate both innate and adaptive immunity, as well as transcription factors that influence cellular phenotype and specific antiviral responses. For example, APOBEC3 is a cytidine deaminase that inhibits human immunodeficiency virus (HIV) replication in macrophages, and IRF1 is a transcription factor involved in both IFN-dependent and IFN-independent antiviral immunity [66,78,79,80,81].

## 4. Functional Diversity of IFNs

Despite their high degree of sequence homology, shared receptor, and similar mechanisms of action, type I IFN subtypes can exhibit significant differences in their potency against various viruses, antiproliferative activity, and ability to activate immune cells [37,82,83]. For example, IFN-β induces a markedly stronger antiproliferative response than IFN-α [37,84,85,86]. Furthermore, IFN-α6 and IFN-α14 demonstrate higher antiviral activity against hepatitis B virus (HBV) and HIV compared to IFN-α2 [87,88,89,90]. Similarly, increased expression of IFN-α13 and IFN-β in colon tumor cells upregulates major histocompatibility complex class I (MHC I) expression and suppresses tumor growth in vivo [91].

The underlying reasons for these functional differences among IFN-α subtypes remain unclear [90]. One hypothesis suggests that IFN-α subtypes prolong and enhance the overall IFN response, providing redundant but quantitatively distinct signaling tiers due to their varying receptor affinities, rather than each subtype possessing a unique function [66]. Conversely, it is hypothesized that the functional differences among IFN subtypes are primarily attributable to their differential affinity for the IFNAR receptor [37,92].

ISGs can be categorized into two groups: the first comprises highly sensitive genes activated by minimal IFN concentrations, while the second requires several hundred-fold higher IFN concentrations for activation. Transcriptomic analyses have shown that genes responsible for direct antiviral activity (e.g., Mx1, PKR, OAS2) typically belong to the first group, whereas genes associated with cell proliferation, chemokine activity, and inflammatory processes (e.g., IL6, CXCL11, TRAIL) often constitute the second group [37,93,94,95,96,97,98,99].

Thomas et al. experimentally demonstrated that IFN-I mutations that reduce receptor-binding affinity diminish both antiviral and antiproliferative potency and attenuate the functional differences between IFN [83]. Notably, mutant variants of IFN-α2 (YNS) and IFN-ω (K152R), which exhibit high affinity for the IFNAR receptor, show a significant increase in antiproliferative activity without a corresponding enhancement of antiviral activity [83]. Thus, the functional specificity of IFNs may be linked to their distinct receptor affinities and the subsequent activation of divergent downstream pathways [37,90]. An intermediate level of receptor affinity appears sufficient for peak antiviral activity, while a significantly higher affinity is required for the maximal antiproliferative effect [37,100].

Studies by Schlaepfer et al. indicated that the biological differences between type I IFNs are quantitative rather than qualitative [101]. The IFN subtypes that most effectively neutralize an infection in vitro can vary depending on the pathogen [89,102,103,104]. However, these differences can be overcome by increasing the IFN dose [89,101]. Although IFN-α subtypes exhibit varying efficiencies in different cellular processes, they do not elicit fundamentally distinct biological responses when administered at sufficiently high concentrations [101]. Differences in antiviral function among IFNs are apparent only at lower doses and converge at doses above 100 pg/mL, with the exception of IFN-α1, which requires doses above 1000 pg/mL [101]. It is important to note that the effector response to IFN can be further modulated by cell type, timing, and the local cytokine milieu [90,105].

## 5. IFNs in Cancer Therapy

Type I IFNs are widely used for therapeutic purposes to treat viral infectious diseases such as chronic hepatitis B and C, HIV, simian immunodeficiency virus (SIV), and lymphocytic choriomeningitis virus (LCMV) [106,107,108,109,110,111,112,113,114,115], as well as neuroinflammatory and oncological diseases, including melanoma, renal cell carcinoma, leukemia, and lymphoma [116,117,118,119].

IFN-α is effective in combating tumors during the elimination phase by enhancing both adaptive and innate immunity. However, in the equilibrium phase, its action starts to shift from anti-tumor to pro-tumor. This occurs because tumor cells that survive the elimination phase due to IFN-α and, consequently, its entire signaling pathway disregulation [7]. This disrupts the expression of STAT1, STAT2, IFNAR, and JAK1 and also leads to the epigenetic suppression of genes involved in the IFN-α signaling pathway. This scenario allows the tumor to grow, spread, and evade immune responses, making its eradication significantly more difficult [120].

Recombinant forms of IFN-α, one of the first cytokines introduced into clinical oncology practice, have remained important tools in the treatment of a number of malignant oncological diseases for decades [7]. Despite the revolutionary emergence of targeted therapies and ICIs, interest in IFN-α has not faded but transformed. Its role is currently being reconsidered in the context of adjuvant regimens, combination therapy, and the use of longer-acting pegylated forms with improved pharmacokinetic characteristics.

Two main recombinant forms of IFN-α, differing in amino acid sequence, are used in modern clinical practice: IFN-α2a and IFN-α2b. Each of these forms is available as both short-lived and pegylated variants, creating a complex of therapeutic possibilities with different pharmacokinetic profiles. The short-lived forms, which include IFN-α2a (Roferon-A, Hoffmann-La Roche Inc., Basel, Switzerland) and IFN-α2b (Intron-A, Merck Sharp & Dohme LLC, NJ, USA), are characterized by rapid clearance from the systemic circulation, requiring frequent (from three times a week to daily injections) administration. In contrast, the pegylated forms, represented by peginterferon α2a (Pegasys, Pharmaand GmbH, Vienna, Austria) and peginterferon α2b (Sylatron/Pegintron, Schering Corporation, Berlin, Germany), are covalent conjugates of IFN with inert polyethylene glycol (PEG) [121] (Table 1). This modification of the molecule significantly increases the drug’s half-life, ensuring stable plasma concentration and reducing injection frequency to once a week. To reduce the toxicity of IFNs and increase their efficacy, numerous approaches besides conjugation with PEG have been proposed, including hybridization with albumin [122]. Chemical modification increases the IFN half-life in the body and prolongs the exposure of tumor cells to high concentrations of IFN, since interferons are crucial for the maturation of DCs and the conversion of naive T-lymphocytes into effector cells. Due to pharmacokinetic differences, drugs based on recombinant IFNs have found application in a wide range of pathological conditions, from the treatment of viral hepatitis B and C and condylomata acuminata to various oncological diseases, such as chronic myelogenous leukemia, renal cell carcinoma, melanoma, and lymphoma [71,113,123,124].

According to current data, IFN therapy is associated with the development of polymorphic adverse effects, which can be classified by the occurrence time and systemic localization [125]. In the acute treatment phase, the most characteristic developments are flu-like syndromes, including fever, chills, myalgia, and arthralgia, often accompanied by gastrointestinal disturbances—nausea, vomiting, and diarrhea [126]. Dose-dependent neurological complications, manifesting as confusion, lethargy, and drowsiness, are regularly described. Chronic toxic effects (such as pronounced asthenic syndrome and an increased risk of clinically significant depressive states) appear as a result of prolonged therapy [127,128]. Hematological toxicity is characterized by the development of myelosuppression with a significant decrease in blood cell levels, while hepatotoxicity manifests as elevated liver transaminases and impaired liver function [129]. These effects are associated with the large size of IFN molecules, their instability, pleiotropic action, and short half-life [130,131,132]. This spectrum of adverse events, systematically documented in clinical studies, continues to be a key factor limiting the therapeutic use of IFNs in modern oncological practice [125].

We analyzed the role of IFN-α, used both as monotherapy and in combination with other drugs, in oncology by studying the completed Phase III and IV clinical trials registered in the ClinicalTrials database (accessed on 10 October 2025). The vast majority of clinical trials were focused on four main oncological conditions: melanoma, renal cell carcinoma (RCC), chronic myeloid leukemia (CML), lymphoma, and osteosarcoma (Table 1).

### 5.1. IFN- α2b

IFN-α2b is the most studied IFN in the context of adjuvant therapy for melanoma. A large study (NCT00004196, *n* ≈ 3000) laid the foundation for the use of the high-dose Intron-A regimen, demonstrating a significant improvement in relapse-free survival (RFS) in patients with high-risk melanoma. The EADO 2001/CMII study (NCT00221702, *n* = 898) directly compared Sylatron (peginterferon-α2b) with non-pegylated Intron-A in patients with stage II melanoma. The study results demonstrated comparable efficacy of pegylated IFN in terms of primary endpoints, combined with a significant improvement in the tolerability profile, making it the preferred choice for long-term therapy [133]. The current relevance of Intron-A continues to be evaluated compared to modern immunotherapeutic agents. In the ongoing S1404 study (NCT01274338, *n* = 1673), a direct comparison of the efficacy of high-dose Intron-A and ipilimumab (anti-CTLA-4) in adjuvant therapy for patients with high-risk melanoma is being conducted. The results of this study are crucial for determining the place of IFN in the new therapeutic reality.

### 5.2. IFN- α2a

IFN-α2a played a key role in the pre-targeted therapy era for RCC. A large Phase III study (NCT00738530, *n* = 649) evaluated combination therapy with bevacizumab—a monoclonal antibody that suppresses angiogenesis—in combination with Roferon-A as first-line therapy in patients with metastatic RCC. The results showed a statistically significant improvement in the median progression-free survival (PFS) in the combination group compared to IFN monotherapy (10.2 vs. 5.4 months; *p* < 0.001), confirming the synergy of anti-angiogenic and immunomodulatory approaches [134].

A promising direction is the use of peginterferon α2a in combination with second-generation tyrosine kinase inhibitors for CML. In a Phase III study (NCT02201459, *n* = 200), patients with newly diagnosed chronic-phase CML received nilotinib in combination with Pegasys. Preliminary data indicate that the combination regimen significantly increased the rate of achieving a deep molecular response (MR4.5), measured by the reduction of BCR-ABL transcripts, after 12 months of therapy compared to nilotinib monotherapy, without a significant increase in toxicity. In the SPIRIT study (NCT00219739) (*n* = 789), the combination of imatinib (a specific tyrosine kinase inhibitor) with IFN-α2a demonstrated an increased rate of MR4.5 in patients with chronic myeloid leukemia [135]. This indicates potential synergy and the possibility of using the immunomodulatory properties of IFN to achieve a deeper molecular response in a certain category of patients.

The general trend shows that the predominant part of modern research is focused on studying recombinant IFNs in combination with other drugs, primarily monoclonal antibodies. The evolution of its application is well traced in various oncological areas.

High-dose IFN-α has been widely used in melanoma patients for many years. Meta-analyses of clinical trials from the last decade, including data from a large number of studies, showed that the rates of improvement in relapse-free survival and overall survival are borderline in terms of statistical significance but are accompanied by a large number of side effects [136,137,138,139,140]. Based on the results of recent and ongoing randomized studies, IFN-α has been displaced by targeted therapy and ICI options [141,142,143,144]. Melanoma experts from the National Comprehensive Cancer Network (NCCN) consider targeted ICIs (aPD-1, aCTLA-4) to be more effective and better tolerated than IFN-α and therefore no longer recommend it for the adjuvant treatment of cutaneous melanoma [145].

According to NCCN guidelines, if treatment for chronic myeloid leukemia is necessary during pregnancy, it is preferable to start with IFN therapy. Most data on the use of IFNs during pregnancy come from patients with essential thrombocythemia. The use of pegylated IFNs in combination with second-generation tyrosine kinase inhibitors is also being studied as a potential strategy [146,147,148]. With earlier use, pegylated IFN-α2a may preserve molecular remission after discontinuation of tyrosine kinase inhibitors [145].

According to NCCN guidelines, the combination of IFN-α and the nucleoside reverse transcriptase inhibitor (zidovudine) is first-line therapy for patients with T-cell leukemia at all stages of the disease—indolent, chronic, and acute. Upon achieving a treatment response, it is recommended to continue this therapy [145]. For the treatment of kidney cancer using IFN, no clinical guidelines are presented by NCCN.

Thus, recombinant IFN-α has come a long way from first-line standards to specialized tools in the arsenal of a modern oncologist. While their standalone use has narrowed, their efficacy remains clinically significant. Pegylated forms have retained their niche for use as adjuvant therapy for some forms of leukemia. The most promising prospects are associated with the integration of pegylated IFNs into combined treatment protocols, where their immunomodulatory potential can be fully realized without excessive toxicity.

### 5.3. IFN-β

In Japan, recombinant IFN-β (Feron, Toray Ltd.) is used for the adjuvant treatment of stage II/III melanoma. In the Phase III clinical trial J-FERON (UMIN000017494, *n* = 240), the efficacy of recombinant IFN-β injected directly into the postoperative wound area was evaluated compared to surgical treatment alone [149,150].

### 5.4. IFN-γ

A promising direction for the use of the drug Actimmune (Horizon Therapeutics Ireland DAC), based on recombinant IFN-γ1b, is its use in the context of allogeneic hematopoietic stem cell transplantation (allo-HSCT) to enhance the graft-versus-leukemia (GVL) effect in patients with relapsed acute myelogenous leukemia and myelodysplastic syndrome. The use of allogeneic donor cells is associated with the risk of graft-versus-host disease [151]. A phase I clinical trial (NCT04628338, *n* = 8) demonstrated that monotherapy with IFN-γ1b followed by donor lymphocyte infusion is safe and is associated with the achievement of complete remission in some patients with relapsed acute myelogenous leukemia and myelodysplastic syndrome after allo-HSCT [152]. The encouraging results formed the basis for a phase II clinical trial (NCT06529731, *n* = 45).

## 6. IFN-Based Gene Therapy for Cancer

The therapeutic effect of recombinant IFNs remains limited, and increasing attention is being paid to treatment strategies based on gene and cell therapy. These approaches are attempting to overcome the fundamental shortcomings of recombinant IFNs, which have a short half-life [130,131,132], requiring frequent injections to maintain a therapeutic effect. At the same time, gene therapy and cell-based drugs enable the production of the IFN protein over a long treatment period, creating a stable and physiologically more natural concentration of the cytokine.

**Figure 4:**
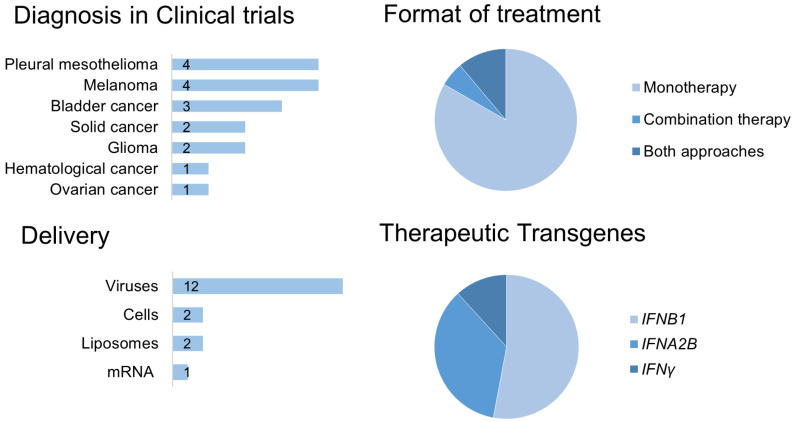
Diagram of clinical trials utilizing IFN-based gene therapy for cancer treatment.

Seventeen clinical trials (CTs) utilizing the delivery of IFN as gene therapy drugs for the treatment of malignant neoplasms were analyzed in this review. The vast majority of CTs, with one exception, consider the use of IFNs as a monotherapy (Figure 4). In only two CTs did researchers, in addition to monotherapy, also evaluate the use of IFN-based gene therapy drugs as part of combination therapy (NCT03710876) [153]. Nearly half of the CTs listed in Table 2 are aimed at the therapy of pleural mesothelioma and melanoma; the use of IFNs for the treatment of bladder cancer is also noteworthy, with Table 2 including results from Phase I, II, and III CTs. The remaining CTs investigate approaches for the treatment of glioma, hematological and solid tumors, and ovarian cancer.

A virus-mediated IFN delivery (70%) was the most popular; 11% of CTs used either autologous tumor cells or plasmid DNA liposomes, and only one study used in vitro transcribed mRNA without a delivery system. In the overwhelming majority of the analyzed CTs, the delivery of various type I IFN-coding sequences is used, which is likely associated with potential concerns about the biological activity of type II IFN (IFN-γ directly affects the intratumoral CD8 T-cells, limiting anti-tumor responses [154]).

Four Phase I clinical trials for the treatment of melanoma employed autologous tumor cells genetically modified to express IFN-γ via retroviral transduction, liposomes with plasmid DNA coding for the *IFNB1*, and a modified vesicular stomatitis virus (VSV) engineered to express IFN-β and tyrosinase-related protein 1 (VSV-IFNβ-TYRP1) [155,156,157,158].

IFN-γ delivery into tumor cells via retrovirus ensures stable protein expression. Subsequent subcutaneous immunization with irradiated autologous tumor cells containing the retrovirus-delivered IFN-γ enhances the humoral immune response post-immunization and further stimulates the presentation of tumor antigens to the patient’s immune system. This contributes to the elimination of the tumorigenicity of the cancer cells through the activation of anti-tumor responses. This effect is likely due to the direct stimulatory action of IFN-γ on macrophages, B-cells, and T-cells. Furthermore, IFN-γ indirectly enhances humoral immunity. There is data showing a positive correlation between the humoral immune response and survival in patients immunized with a mix of allogeneic melanoma cells and Bacillus Calmette-Guérin (BCG) [159].

As shown in Table 2, the results of CTs for melanoma therapy using autologous tumor cells modified to secrete IFN-γ demonstrate a limited therapeutic effect. In the first study relying on genetically modified autologous tumor cells, only 65% of patients were able to complete the full treatment protocol. An objective response (tumor regression or significant reduction in tumor volume) was recorded in 31% of patients, while 60% of subjects responded to immunization (predominantly an IgG response) [155]. In the second study, an increase in overall survival rates was observed in 47% of participants (8 out of 17 cases) [156], with 12% of patients showing a complete response to treatment at the site of intratumoral injection for metastatic melanoma.

Gene therapy for cancer based on the IFN-γ delivery by autologous tumor cells can alter tumor antigen expression and induce a localized immune response. However, in patients with severe, progressive disease stages and an expected survival of less than one year, it can be difficult to assess the clinical effects of a systemic response. Therefore, it is quite probable that the demonstrated moderate therapeutic efficacy may be enhanced in patients with a less advanced stage of the disease. Another strategy for the treatment of melanoma is based on the delivery of the *IFNB1* into the tumor. Melanoma cells often exhibit mutations/epigenetic suppression of this gene’s transcription [160]. Autocrine IFN secretion, as opposed to exogenous, was found to significantly suppress the proliferation of melanoma cells [161]. Delivering the *IFNB1* into melanoma cells with its defective expression may increase these cells’ susceptibility to *IFNB1*, which exerts a dose-dependent anti-proliferative and apoptotic effect on melanoma cells.

Uveal melanoma differs from cutaneous melanoma in its low overall survival rate in the metastatic form, reaching as low as 8% [162]. Moreover, it is less sensitive to ICI therapy [163,164], underscoring the need for the development of new therapeutic approaches and their combinations. Relatively recently, Phase I CT data for the therapy of uveal melanoma with VSV-IFNβ-TYRP1 were published [158]. In this study, the therapeutic effect depends largely on the use of the oncolytic VSV [165,166], while the introduction of additional transgenes into its genome influences the additional efficacy and safety. Although no clear objective responses to VSV-IFNβ-TYRP1 therapy were observed, the authors emphasize the importance of a combination regimen with ICIs: in 2 patients from the trial, the efficacy of the ICI therapy course following VSV-IFNβ-TYRP1 was confirmed both clinically and by T-cell reactivity (Table 2).

An alternative strategy for *IFNB1* delivery into tumor cells is the use of non-viral delivery systems. In particular, the use of liposomes with plasmid DNA coding for the *IFNB1* gene is an in vitro validated strategy that achieves a high level of secreted protein 48 h after transfection. Therapy using liposomes carrying the *IFNB1* gene led to the complete regression of tumor lesions in 1 out of 5 patients [157].

The results summarized in Table 2 demonstrate that a similar narrative regarding the application of type I IFNs has been observed for other tumor types, such as pleural mesothelioma and gliomas (NCT03710876) [167,168,169,170]. Pleural mesothelioma is a rare, aggressive form of cancer arising from the mesothelial cells lining internal organs. Diagnosing this cancer type remains challenging, and the disease itself has limited treatment options and a poor prognosis. The current standard of care for mesothelioma involves surgery, radiation therapy, and chemotherapy [171]. The efficacy of immunotherapy using ICIs remains questionable, although it may benefit some patients previously treated with chemotherapy [172].

For the treatment of pleural mesothelioma, adenoviruses delivering two different subtypes of type I IFNs have been employed: IFN-β (rAd-IFNβ) and IFN- α2b (rAd- IFNα2b). While numerous studies utilize adenoviruses as oncolytic viruses [173], in the selected CTs, they are described specifically as vectors for transgene delivery.

**Table 2:** Gene therapy drugs based on IFNs.

Diagnosis	Clinical Trial Phase	Cohort (Sample Size, Age)	Intervention Name	Therapeutic Transgene	Delivery	Dose, Route, And Regimen	Format of Therapy	Therapeutic Outcome by RECIST Criteria	Adverse Events
Melanoma	Phase I [155]	*n* = 20, aged 18–65 and older	IFNγ gene-modified autologous tumor cells	IFN-γ	Autologous cells	ID, 6 injections (2 × 10^6, 6 × 10^6, 18 × 10^6 cells)	Monotherapy	CR 10%, PR 10%	No information is available
	Phase I [156]	*n* = 17, aged 34–86	IFNγ gene-modified autologous tumor cells	IFN-γ	Autologous cells	IT, 6 injections (2 × 10^6, 6 × 10^6, 18 × 10^6 cells)	Monotherapy or combined with IL-2	SD 23.5%, Median OS: 150 days (single-dose injection) and >1.5 years (multiple-dose injections)	No Grade 3–4 TEAEs
	Phase I [157]	*n* = 5, aged 33–73	IAB-1	*IFNB1*	Liposomes	IT, dose: 30 μg DNA, three times per week for 2 weeks.	Monotherapy	PD 60%, SD 20%, CR 20%	None observed
	Phase I [158]	*n* = 12, median age 69.5	VSV-IFNβ-TYRP1	*IFNB1* *TYRP1*	VSV	IT, dose 3 × 10^7 TCID50 and IV, dose 1 × 10^10 TCID50 or 3 × 10^10, 1 × 10^11, 3 × 10^11	Monotherapy	SD 33.33%, PD 66.67%	Grade 4 TEAEs (*n* = 4); grade 3 TEAEs (*n* = 3); grade 1/2 TEAEs (all others).
Pleural mesothelioma	Phase I [167]	*n* = 10, aged 40–80	Ad.IFN-β	*IFNB1*	Adenovirus	IV, single dose, 9 × 10^11 to 3 × 10^12 vp.	Monotherapy	SD 40%, PD 60% 60 days after the start of treatment, PR/SD 40% 6 months after the start of treatment	Grade 1–2 TEAEs (*n* = 3); grade 3 TEAEs (*n* = 2).
	Phase I [171]	*n* = 17, aged 50–87	BG00001	*IFNB1*	Adenovirus	IP, two doses of 3 × 10^11, 1 × 10^12, or 3 × 10^12 vp every 14 days or 1.5 × 10^12 or 3 × 10^12 vp every 7 days	Monotherapy	PR 5.9%, SD 11.7%, PD 52.9%, 7 patients had an OS of >18 months	Grade 1–2 TEAEs (*n* = 17); grade 3 lymphopenia (*n* = 12); grade 4 pericardial tamponade (*n* = 1).
	Phase III (NCT03710876)	*n* = 27, aged 18–65 and older	rAd-IFN	*IFNA2B*	Adenovirus	IV, single dose, 3 × 10^11 vp.	Combined with Celecoxib and Gemcitabine	CR, PR, or SD 70.4%, Median OS after 42 months: 25.9 months (11.5–43.1)	Grade ≥ 3 TEAEs (*n* = 10); grade 1–2 TEAEs (*n* = 27).
	Phase I (NCT01212367) [168]	*n* = 9, aged 18 and older	Ad.IFN-α2b	*IFNA2B*	Adenovirus	IP, one or two doses of 1 × 10^12 or 3 × 10^11 VP	Monotherapy	PD 44.4%, SD 44.4%, CR 11.1%	Grade 1–2 TEAEs (*n* = 9), grade 3 TEAEs—flu-like symptoms requiring dose reduction (*n* = 2).
Glioma	Phase I [169]	*n* = 5, aged 28–64	pDRSV-IFN-β	*IFNB1*	Liposomes	IT, 3 (on days 14, 21, 28) or 5 (on days 14, 17, 21, 24, 28) injections, dose: 30 μg DNA.	Monotherapy	CR 40%, SD 60% 10 months after, Median OS: 9 ± 2.7 and 17 ± 4.5 months	No information is available
	Phase I [170]	*n* = 11, aged 18 and older	Ad.hIFN-β	*IFNB1*	Adenovirus	IT, dose 2 × 10^10, 6 × 10^10, or 2 × 10^11 VP	Monotherapy	PD 100% within 4 months after the start of treatment	Grade ≥ 3 TEAEs cerebellar inflammation/necrosis (*n* = 1); other AEs, not drug-related.
Solid cancer	Phase I (NCT02923466) [174]	*n* = 20, aged 18 and older	Voyager-V1 (VV1)	*IFNB1*	VSV	IV, dose 1.7 × 10^10 TCID50 or 1.0 × 10^11 TCID50.	Monotherapy	No information is available	Acceptable safety profile. No Grade 4 IRRs or deaths were observed.
	Phase I [153]	*n* = 21, aged 32–89	SAR441000	IFN-α-2b, IL-12, GM-CSF, and IL-15	single-stranded, 5′-capped mRNA	IT, once weekly for 4 weeks, dose: 8 ng to 4000 ng.	Monotherapy	SD—19.0%, PD—61.9%.	Grade 1–2 TEAEs (*n* = 21); Grade 3 TEAEs (*n* = 9).
		*n* = 15, aged 32–89				IT, once weekly for 3 weeks, dose: 200 ng to 4000 ng; and Cemiplimab, IV, dose: 350 mg.	Combined with Cemiplimab	CR—6.7%, PR—20.0%, SD—73.3%, Median PFS: 2,0 months (90% CI: 1.02–2.12).	Grade 1–2 TEAEs (*n* = 15); Grade 3 TEAEs (*n* = 1).
		*n* = 41, aged 32–89				IT, once weekly for 3 weeks, dose: 4000 ng; and Cemiplimab, IV, dose: 350 mg.		CR—4.9%, PR—24.4%, SD—58.5%, NE—12.2%, ORR 4.9% (90% CI: 0,9–14,6) after a median observation time of 8.9 months. OS: 29.3% (90% CI: 17.8–43.1); median PFS: 8.9 months (90% CI: 4.99–NR); median OS: 2.1 months (90% CI: 1.95–2.28).	Grade 1–2 TEAEs (*n* = 33); grade ≥ 3 TEAEs (*n* = 6).
Hematological cancer	Phase I (NCT03017820) [175]	*n* = 30, aged 18 and older	Voyager-V1 (VV1)	*IFNB1*	VSV	IV, dose: 5 × 10^9 to 1.7 × 10^11 TCID50.	Monotherapy	No information is available	No information is available
Ovarian cancer	Phase I [176]	*n* = 1, aged 47	BG00001,	*IFNB1*	Adenovirus	IP, dose: 9 × 10^11 VP	Monotherapy	PD 100%	Grade 1–2 TEAEs.
Bladder cancer	Phase I [177,178]	*n* = 17, aged 18 and older	rAd-IFNα2b/Syn3	*IFNA2B*	Adenovirus	IVes, dose: 3 × 10^9 to 3 × 10^11 VP	Monotherapy	CR 43%, Remission of the disease 41.18%.	Grade 1–2 TEAEs (*n* = 17).
	Phase II NCT01687244 [177,179]	*n* = 43, aged 62–81	rAd-IFNα2b/Syn3	*IFNA2B*	Adenovirus	IVes, dose 1 × 10^11 or 3 × 10^11 VP	Monotherapy	The 12-month RFS rate was 25%	Grade 1–2 TEAEs (*n* = 39); grade 3 TEAEs (*n* = 9).
	Phase III NCT02773849 [177,180]	*n* = 157, aged 66–77	Nadofaragene firadenovec (rAd-IFNα2b/Syn3)	*IFNA2B*	Adenovirus	IVes, dose 3 × 10^11 VP. Redosing at 3, 6, and 9 months in the absence of high-grade relapse.	Monotherapy	The 3-month CR rate was 53%, and the 12-month CR rate was 24%. The 3-month RFS was 73% among patients with HGTa/T1 tumors, and the 12-month RFS was 44%.	Grade 1–2 TEAEs (majority of patients), grade 3–4 TEAEs (*n* = 6).

PD—Progressive Disease, SD—Stable Disease, NE—Not Evaluable, CR—Complete Response, PR—Partial Response, ORR—Objective Response Rate, CI—Confidence Interval, median PFS—Progression-Free Survival, OS—Overall Survival, relapse-free survival—RFS, complete remission—CR, Not Reached—NR, TEAEs—treatment-emergent AE, IT- Intratumoral injection, IV—Intravenous injection, IVes—intravesical injection, ID—intradermal injection, IP—Intrapleuralis injection.

In a Phase I clinical trial using rAd-IFNα2b, disease stabilization or partial regression of tumor foci was recorded in 55.6% of patients (5 out of 9 cases) [168]. One of these patients exhibited a systemic anti-tumor effect, manifested by the regression of distant metastatic lesions not directly exposed to the drug, illustrating a hallmark feature of immunotherapeutic approaches. The efficacy of rAd-IFNα2b was also confirmed in a Phase III trial (NCT03710876), where disease control was achieved in 70.4% of patients (19 out of 27 cases).

Studies using rAd-IFNβ demonstrated disease control in 30% of patients (3 out of 10 subjects) [171], tumor stabilization or regression in 40% of cases (4 out of 10 patients) [168], and a temporary positive response in one patient followed by subsequent tumor progression [176].

Given that the viral vectors used in these trials were nearly identical and the biological activity of IFN-α and IFN-β is very similar, it was anticipated that the outcomes for this therapeutic approach in pleural mesothelioma would be comparable. However, the delivery of IFN-α2b resulted in much higher pleural concentrations of IFN at equivalent or even lower vector doses compared to IFN-β delivery [168]. The reason for this enhanced efficacy may be related to greater intrapleural stability of the IFN-α2b mRNA and/or protein. It is important to note, however, that the described clinical trials remain exploratory in nature, investigating new approaches for a disease characterized by a high mortality rate (median survival for stage 1 is 21 months, and for stage 4 it is 12 months [181]).

Gliomas represent a group of heterogeneous primary brain tumors that vary in their degree of malignancy, histology, and genomic alterations. The median overall survival for patients diagnosed with glioma, based on clinical trial data following radiotherapy, is 12.1 months [182]. One of the most challenging aspects in developing effective treatments for gliomas is the ability of therapeutic agents to reach the tumor site at sufficient therapeutic concentrations due to the presence of the blood-brain barrier (BBB) [183]. Consequently, gene therapy-based approaches are particularly promising for patients with diagnosed glioblastoma.

According to Table 2, in a CT of a liposome-based drug carrying the *IFNB1* gene, patient survival was reported at 17 ± 4.5 months, with a more than 50% reduction in tumor size observed in 40% of patients (2 out of 5 cases) [169]. Another study investigating glioma therapy with an adenovirus delivering IFN-β (rAd-IFNβ) reported a median survival of 4.1 months (or 17.9 weeks) [170]. These selected CTs illustrate the promise of IFN-β-based gene therapy. However, it is important to note that when using the now-classic adenoviral delivery system, the viral DNA was detected in the blood and nasal swabs in a dose-dependent manner, although a dose-dependent increase in tumor cell apoptosis was reproducibly observed in post-treatment biopsies.

A notable finding from the CT using liposomal delivery of the IFN-β gene is that this method appears to exert an anti-angiogenic effect. This is particularly important in glioblastoma therapy, as these tumors are among the most vascularized malignancies, a feature that correlates with their biological aggressiveness, malignant grade, and clinical recurrence [184]. Therefore, the choice of the delivery system remains no less critical than the transgene being delivered.

The advent of gene therapy approaches using IFNs promised to overcome the systemic toxicity of recombinant proteins and create potent local anti-tumor immunity. However, on the way to clinical success, these drugs faced the problem of low therapeutic efficacy, especially in the treatment of aggressive tumors such as melanoma, glioma, and pleural mesothelioma. The waning interest in conducting CTs is largely associated with two key factors: competition with more effective treatment strategies and moderate therapeutic efficacy when used in severely ill patients [185,186,187]. Phase I studies enrolled patients with advanced, refractory forms of cancer who had exhausted all standard treatment options [155,156,157,158]. For instance, injection of the drug into tumor nodules in metastatic melanoma, even when a local response occurs, does not lead to long-term stabilization of the systemic disease [157,158]. Thus, the problem may lie not in the erroneousness of the concept but in its application in the most unfavorable conditions—against the most challenging tumors in the most severely ill patients, amidst the emergence of more effective types of therapy.

More recent research focusing on the development of type I IFN-based gene therapy drugs is aimed at combating solid tumors (Table 2). The drug VV1 (VSV expressing human IFN-β and the sodium iodide symporter) demonstrated an acceptable safety profile based on the results of a phase I clinical trial with systemic administration in patients with advanced inoperable and metastatic solid tumors [174]. There is also data from another CT using VV1 for the therapy of refractory recurrent hematological tumors [175]. The systemic format of VV1 therapy was not chosen by chance: when delivered via such a route to patients with metastatic solid and hematological tumors, VV1 infects tumor cells and macrophages in the lymph nodes and spleen, leading to the release of tumor antigens and IFN-β, which activates and enhances cellular anti-tumor immunity. Another novel approach is the use of mRNA technology for the delivery of a cytokine combination in advanced solid tumors [153]. Intratumoral administration of the drug SAR441000 (a mix of four mRNAs encoding IL-12, single-chain IFN-α-2b, GM-CSF, and the IL-15 sushi domain) alone or in combination with cemiplimab demonstrated anti-tumor activity in locoregional lesions, while no significant effects were observed in patients with advanced solid tumors previously treated with ICI therapy. SAR441000 monotherapy showed isolated tumor reduction in injected lesions and stable disease control in several patients; however, the efficacy itself was insufficient. Researchers suggest that the lack of significant objective responses might be associated with the use of mRNA cytokines formulated in saline without a delivery system, which could lead to lower than expected cytokine concentrations in the tumor.

As shown in Table 2 in a Phase I CT of IFN-β therapy delivered by an adenovirus, one patient was included whose ovarian cancer had progressed despite chemotherapy and hormonal treatment [176]. Twenty-four hours after the injection of 9 × 10^11 viral particles (VP) delivered via a tunneled pleural catheter, a rapid and marked elevation of intrapleural IFN-β was observed, which declined to undetectable levels over 1 week. Although tumor regression in abdominal foci was observed 2 months after treatment, follow-up studies at 4 months showed disease progression with the appearance of new lesions and an increase in the size of existing ones.

The most promising niche for the application of gene therapy drugs based on type I IFN is the treatment of bladder cancer, which ranks tenth in the incidence of malignant neoplasms [188] (Table 2). The current standard of treatment for non-muscle-invasive bladder cancer (NMIBC) is transurethral resection followed by intravesical therapy with BCG [189]. Previously, the lack of effective treatment options for non-invasive bladder cancer unresponsive to BCG stimulated drug development in this area [190,191]. Furthermore, studies have shown that intravesical administration of the IFN-α protein was ineffective, mainly due to insufficient exposure and temporary availability of IFN-α after instillation into the bladder [192]. CTs of the drug nadofaragene firadenovec (Adstiladrin^®^, Ferring Pharmaceuticals, Kastrup, Denmark), created based on an adenoviral vector with the *IFNα2b* gene (rAd-IFNa2b), demonstrated high efficacy in the treatment of bladder cancer—according to phase III CT results, 53% of patients achieved complete remission at 3 months, with 24% maintaining a response to therapy for 12 months [177,180]. The treatment showed a significant response rate and an acceptable safety profile, after which, in 2022, the FDA approved the use of this drug in adult patients with NMIBC who are unresponsive to BCG therapy. The importance of the auxiliary component Syn3 in bladder cancer therapy should be noted, as it overcame the difficulties of effective adenoviral transduction of urothelial cells, leading to high and sustained concentrations of IFNα in the urine and tumor regression [177]. This gene therapy for this type of bladder cancer is the first gene therapy method approved by the FDA for the treatment of urological diseases.

## 7. Discussion

The therapeutic application of IFN-α in cancer, including hairy cell leukemia, lymphoma, renal cell carcinoma, and melanoma, was one of the first successes of immunotherapy [193,194,195]. However, the low response rate in many solid tumors and significant toxicity led to this therapy being overtaken by other, more effective approaches [196]. A turning point was a 2020 Phase III trial that demonstrated the superiority of ipilimumab (an anti-CTLA-4 antibody) over high-dose IFN-α2b, shifting the treatment paradigm for operable melanoma towards ICI immunotherapy [193,197]. Nevertheless, interest in IFNs has persisted due to several factors. First, IFNs may retain a therapeutic niche in cancers with low response rates to ICIs, such as uveal melanoma and ovarian cancer, as shown in some CTs [158,198,199]. Second, although IFN monotherapy shows limited efficacy, its potential is revealed in combination therapy. For instance, CTs confirm the successful combination of IFNs with chemoradiation therapy [200,201,202,203] and immunotherapy [204]. Type I IFNs significantly enhance the efficacy of immunostimulatory agents and ICIs [205,206], as demonstrated in a number of preclinical studies. In the work of Zhu et al., the combination of Peg-IFNα with PD-1 blockade significantly enhanced tumor T-cell infiltration, increased the efficacy of anti-PD-1 antibodies, and improved mouse survival in a hepatocellular carcinoma model compared to anti-PD-1 antibody monotherapy [207]. Similar results were obtained in a mouse melanoma model, showing that IFN-β stimulates the expression of the ligands CCL5 and CXCR3 and induces tumor infiltration by T-lymphocytes. This, in turn, enhances the efficacy of treatment with anti-PD-L1 antibodies [208]. A Phase I clinical trial of the drug SAR441000 also confirmed the promise of combining IFN with a PD-1 inhibitor for the treatment of solid tumors. The combination cohort (mRNA + cemiplimab) showed a higher objective response rate (ORR = CR + PR) of 26.7%, compared to no responses in the monotherapy group [153].

Although IFN as a standalone drug has been superseded by more effective methods, its therapeutic potential might be re-evaluated. One direction that could revive interest in IFN is improving its delivery methods. A limitation for the clinical use of type I IFNs is their short half-life, which necessitates high, non-physiological doses that lead to systemic toxicity. Gene therapy, which provides prolonged local cytokine expression, is considered a promising alternative. For example, a preclinical study [209] showed that intratumoral injection of an adenoviral vector encoding IFNα2b (IACB) suppressed the growth of xenografts, including glioblastoma (U87MG) and leukemia (K562) models; significant growth inhibition was also observed with systemic administration. A key finding was that after a single administration of IACB, circulating IFN was detectable for up to 15 days. This prolonged expression profile maintained an effective IFN concentration over a long period and minimized the need for repeated high-dose administrations. In the study [210], the combination of AdhIFNβ with 5-fluorouracil (5-FU) had a significant therapeutic effect, leading to substantial tumor regression in mice, with complete disappearance of some tumors. In contrast, administration of recombinant IFN-β protein in combination with 5-FU provided no therapeutic advantage. Convincing evidence for the efficacy of adenovirus-mediated IFN delivery is the success of the phase III CT of the drug Nadofaragene firadenovec for bladder cancer, where local delivery of IFNA2B via an adenovirus achieved high efficacy rates: complete remission was achieved in half of the patients (53%), and nearly a quarter (24%) maintained a response for 12 months. The results in patients with HGTa/T1 tumors are particularly impressive: 73% and 44% were free of high-grade recurrence at 3 months and one year, respectively. This example suggests that for other tumor types, targeted IFN delivery could also unlock its therapeutic potential while minimizing systemic toxicity [177,180,211].

Thus, despite the undeniable role of type I IFN signaling in suppressing carcinogenesis and compelling evidence of its efficacy in preclinical models [212,213,214,215,216,217,218,219], its translation into clinical practice remains limited. The therapeutic potential of these cytokines is currently realized only in a narrow niche of cancers.

An additional factor limiting the use of IFNs is the complexity and incomplete understanding of type I IFN signaling in tumor cells. In particular, the functional specialization of individual subtypes, especially the numerous IFN-α genes, and their contribution to anti-tumor immunity remain unclear, despite some data on their differing biological activities and anti-tumor effects [220,221,222].

It is also important to note that anti-cancer therapy often focuses on type I IFNs, while other IFN types do not receive sufficient attention. However, in some cases, using type II IFN or a combination of type I and type II IFNs proves more effective. For instance, the therapeutic potential of type I and type II IFN combination therapy is revealed in a new formulation of IFNs (HeberFERON) which resulted in a higher number of complete responses for patients with basal cell carcinoma and prolongation of survival for patients with glioblastoma and renal cell carcinoma [223]. HeberFERON, a co-formulated interferon product, demonstrated enhanced pharmacodynamic properties, including greater potency and prolonged duration of action, compared to its individual components. This improved profile allows for reduced dose frequency and lower doses, and, hence, a favorable safety and tolerability profile. Clinical trials with HeberFERON are included in the Cuban Registry of Clinical Trials (https://rpcec.sld.cu/en/home, accessed on 24 November 2025). Also, combining type I and type II IFNs is revealed in Phase I clinical trials using autologous monocytes activated by IFNs to treat ovarian cancer. It is known that therapy with ICIs [224] has limited efficacy in ovarian cancer, making the search for new approaches crucial. Preclinical studies in mouse models demonstrated that treatment of monocytes with IFN-α and IFN-γ drives their differentiation into a pro-inflammatory M1 phenotype while suppressing the development of the anti-inflammatory M2 phenotype. Intratumoral injection of these activated monocytes led to a significant reduction in tumor volume and increased survival [198]. These encouraging findings were confirmed in Phase I clinical trials [199]. Therapy with autologous IFN-activated monocytes showed good tolerability and clinical efficacy. In one study (NCT02948426), 2 out of 11 patients achieved a partial response (in one case with a 61% tumor reduction), and in 4 patients the disease stabilized. In another trial [225], a similar partial response was observed in 2 out of 9 patients. The proposed mechanism of action involves the induction of caspase-8-dependent apoptosis by the pro-apoptotic tumor necrosis factor-related apoptosis-inducing ligand (TRAIL), mediated by death receptors 4 and 5 (DR4 and DR5, respectively) on cancer cells. Furthermore, for some cancers, combination therapy with IFN-γ is effective. For example, CT results demonstrated that IFN-γ sensitized myeloid leukemia cells to alloreactive T cell-mediated killing and could be used as maintenance therapy for patients with relapsed acute myeloid leukemia or myelodysplastic syndrome after allogeneic bone marrow transplantation [152,226].

Looking ahead, the primary direction for realizing the anti-tumor potential of IFN involves overcoming two key barriers: systemic toxicity and an insufficient response within the tumor. Solving the first challenge is linked to the development of platforms for targeted delivery of gene therapy drugs, while the second requires a deeper understanding of IFN mechanisms of action and the development of effective combinations with other immunotherapeutic agents.

## 8. Conclusions

Despite the limited application of IFNs in modern oncology, they remain clinically significant therapy options. For example, the pegylated alternatives remain the standard of adjuvant therapy for some types of leukemia and continue to be used in the treatment of melanoma in certain clinical scenarios. At the same time, genetically engineered drugs demonstrated high efficacy against highly specialized oncological diseases, such as bladder cancer. IFN-based cancer therapy offers several promising prospects. First, a combination of IFN subtypes (e.g., HerberFERON) can improve the IFN therapy potency against basal cell carcinomas, glioblastomas, and renal cell carcinomas. Second, new knowledge of the therapeutic potential of less common IFN subtypes (in addition to IFNa2a, IFNa2b, and IFNβ) and their combinations can further boost the IFN clinical niche. Third, continued improvement of delivery methods can help overcome IFN toxicity compared to their systemic administration. Fourth, identification of new biomarkers of sensitivity and tumor subtypes may help target patients most susceptible to treatment. Given the gigantic progress made in the past decade in biomedical research in intratumoral delivery of immunotherapies (including viral vector-mediated delivery), mRNA platforms, and combination therapy, we believe that IFN-based drugs will experience a renewed lease of life, giving hope to cancer patients.

## Figures and Tables

**Table 1:** Use of Recombinant IFN Proteins for Cancer Therapy.

IFN Type	Name and Organization	Treatments	Clinical Trial Identification Number (Phase 3/4) (Year, Enrollment)
IFN-α2a	Roferon-A (Hoffmann-La Roche Inc.)	Chronic Myelogenous Leukemia	NCT02829775 (2008-9) NCT00333840 (2012-1106) NCT00219739 (2014-789)
		Melanoma	NCT02829775 (2008-9) NCT00204529 (2016-901) NCT00002892 (2001-1000)
		Renal Cell Carcinoma	NCT02829775 (2008-9) NCT00738530 (2008-649) NCT00083889 (2008-750) NCT00502034 (2012-310) NCT00002737 (2012-320)
		Lymphoma	NCT01609010 (2010-313)
Peginterferon α2a	Pegasys (Pharmaand GmbH)	Chronic Myelogenous Leukemia	NCT02736721 (2010-41) NCT02829775 (2008-9) NCT00219739 (2014-789) NCT02201459 (2022-200)
		Melanoma	NCT00204529 (2016-901) NCT02829775 (2008-9)
		Renal Cell Carcinoma	NCT02829775 (2008-9)
IFN-α2b	Intron A (Merck Sharp & Dohme LLC, NJ, USA)	Melanoma	NCT00221702 (2010-898) NCT01259934 (2008-855) NCT00002882 (2006-140) NCT00004196 (2007-3000) NCT02506153 (2026-1301) NCT01274338 (2026-1673) NCT00003444 (2003-167)
		Basal Cell Carcinoma	NCT00581425 (2011-49)
		Renal Cell Carcinoma	NCT00072046 (2012-732)
Peginterferon α2b	Sylatron/Pegintron (Schering)	Melanoma	NCT00221702 (2010-898)
		Osteosarcoma	NCT00134030 (2022-1334)
		Chronic Myelogenous Leukemia	NCT00050531 (2015-94) NCT07105319 (2025-65)
IFN-β	Feron, (Toray Ltd, Tokyo, Japan)	Melanoma	UMIN Clinical Trials Registry identification number UMIN000017494 (2015-240)
IFN-γ1b	Actimmune (Horizon therapeutics Ireland dac, Dublin, Ireland)	Acute Myelogenous Leukemia Myelodysplastic Syndrome	Phase 1 and Phase 2 NCT04628338 (2023-8) NCT06529731 (2027-45)

## Data Availability

No new data were created or analyzed in this study. Data sharing is not applicable to this article.

## Abbreviations

The following abbreviations are used in this manuscript:

IFNs	Interferons
BiTE	Bispecific T-cell engagers
CAR-T	T-cell therapy
IFN-I	Type I interferons
IFN-II	Type II IFN
IFN-III	Type III IFNs
ICIs	Immune checkpoint inhibitors
PAMPs	Pathogen-Associated Molecular Patterns
DAMPs	Damage-Associated Molecular Patterns
DNA	Deoxyribonucleic Acid
RNA	Ribonucleic Acid
ATP	Adenosine triphosphate
PRRs	Pattern recognition receptors
TLRs	Toll-like receptors
CLRs	C-type lectin receptors
RLRs	RIG-I-like receptors
NLRs	NOD-like receptors
ALRs	AIM2-like receptors
DCs	Dendritic cells
NK	Natural killer
MyD88	Myeloid Differentiation primary response protein 88
TRIF	TIR-domain-containing adapter-inducing interferon-β
VREs	Virus-responsive elements
IRFs	Interferon regulatory factors
cGAS	Cyclic GMP–AMP synthase
NF-κB	Nuclear factor κB
cGAS–STING	Cyclic GMP–AMP synthase–stimulator of interferon genes
RLR–MAVS	RIG-I–like receptors–mitochondrial antiviral-signaling protein
STAT	Signal transducers and activators of transcription
ISGs	IFN-stimulated genes
MAPK	Mitogen-activated protein kinase
PI3K	Phosphoinositide 3-kinase
IL	Interleukin
IFI16	Interferon Gamma Inducible Protein 16
DDX41	DEAD-box helicase 41
DNA-PK	DNA-Dependent Protein Kinase
MRE11	Meiotic Recombination 11
DAI	DNA-Dependent Activator of Interferon-regulatory factors
IFNGR1	Interferon-Gamma Receptor subunit 1
IFNGR2	Interferon-Gamma Receptor subunit 2
IFNLR1	Interferon-Lambda Receptor 1
IFNAR1	Interferon alpha and beta receptor subunit 1
IFNAR2	Interferon alpha and beta receptor subunit 2
HBV	Hepatitis B virus
MHC I	Major histocompatibility complex class I
Mx1	Myxovirus resistance 1
PKR	Protein kinase R
OAS2	2′–5′-Oligoadenylate Synthase 2
CXCL11	Chemokine (C-X-C motif) ligand 11
TRAIL	Tumor Necrosis Factor (TNF)-Related Apoptosis-Inducing Ligand
SIV	Simian immunodeficiency virus
PEG	Polyethylene glycol
RCC	Renal cell carcinoma
CML	Chronic myeloid leukemia
anti-CTLA-4	anti-Cytotoxic T-Lymphocyte Antigen 4
aPD-1	anti- Programmed cell Death 1
CTs	Clinical trials
BCG	Bacillus Calmette-Guérin
VSV	Vesicular Stomatitis Virus
Ad	Adenovirus
TYRP1	Tyrosinase-Related Protein 1
PD	Progressive Disease
SD	Stable Disease
NE	Not Evaluable
CR	Complete Response
PR	Partial Response
ORR	Objective Response Rate
CI	Confidence Interval
PFS	Progression-Free Survival
OS	Overall Survival
RFS	Relapse-free survival
CR	Complete remission
NR	Not Reached
TEAEs	Treatment-emergent AE
IT	Intratumoral injection
IV	Intravenous injection
IVes	Intravesical injection
ID	Intradermal injection
IP	Intrapleuralis injection
BBB	Blood-brain barrier
GM-CSF	Granulocyte-Macrophage Colony-Stimulating Factor
